# Preoperative Prediction of Lymphovascular Space Invasion in Cervical Cancer With Radiomics –Based Nomogram

**DOI:** 10.3389/fonc.2021.637794

**Published:** 2021-07-12

**Authors:** Wei Du, Yu Wang, Dongdong Li, Xueming Xia, Qiaoyue Tan, Xiaoming Xiong, Zhiping Li

**Affiliations:** ^1^ Department of Radiotherapy, Cancer Center, West China Hospital, Sichuan University, Chengdu, China; ^2^ State Key Laboratory of Biotherapy and Cancer Center, West China Hospital, Collaborative Innovation Center for Biotherapy, Sichuan University, Chengdu, China; ^3^ Department of Radiology, The Affiliated Hospital of Southwest Medical University, Luzhou, China; ^4^ School of Computer Science & Engineering, South China University of Technology, Guangzhou, China; ^5^ Department of Head and Neck Oncology, Cancer Center, West China Hospital, Sichuan University, Chengdu, China; ^6^ Department of Pathology, The Affiliated Hospital of Southwest Medical University, Luzhou, China

**Keywords:** LVSI, radiomics, cervical cancer, diagnostic performance, invasion

## Abstract

**Purpose:**

To build and evaluate a radiomics-based nomogram that improves the predictive performance of the LVSI in cervical cancer non-invasively before the operation.

**Method:**

This study involved 149 patients who underwent surgery with cervical cancer from February 2017 to October 2019. Radiomics features were extracted from T2 weighted imaging (T2WI). The radiomic features were selected by logistic regression with the least absolute shrinkage and selection operator (LASSO) penalty in the training cohort. Based on the selected features, support vector machine (SVM) algorithm was used to build the radiomics signature on the training cohort. Incorporating radiomics signature and clinical risk factors, the radiomics-based nomogram was developed. The sensitivity, specificity, accuracy, and area under the curve (AUC) and Receiver operating characteristic (ROC) curve were calculated to assess these models.

**Result:**

The radiomics model performed much better than the clinical model in both training (AUCs 0.925 *vs.* 0.786, accuracies 87.5% *vs.* 70.5%, sensitivities 83.6% *vs.* 41.7% and specificities 90.9% *vs.* 94.7%) and testing (AUCs 0.911 *vs.* 0.706, accuracies 84.0% *vs.* 71.3%, sensitivities 81.1% *vs.* 43.4% and specificities 86.4% *vs.* 95.0%). The combined model based on the radiomics signature and tumor stage, tumor infiltration depth and tumor pathology yielded the best performance (training cohort, AUC = 0.943, accuracies 89.5%, sensitivities 85.4% and specificities 92.9%; testing cohort, AUC = 0.923, accuracies 84.6%, sensitivities 84.0% and specificities 85.1%).

**Conclusion:**

Radiomics-based nomogram was a useful tool for predicting LVSI of cervical cancer. This would aid the selection of the optimal therapeutic strategy and clinical decision-making for individuals.

## Introduction

Cervical cancer is the fourth most frequently diagnosed cancer and the fourth leading cause of cancer death in women in 2018 worldwide (1). For early-stage disease, both radical hysterectomies with lymph nodes dissection and pelvic radiation therapy with vaginal brachytherapy are equally effective with approximate 5-year survival rates of 87% to 92% (2–4). Following surgery, postoperative radiotherapy (PORT) is indicated for patients with adverse pathologic factors by the National Comprehensive Cancer Network (NCCN) and the 2018 International Federation of Gynecology and Obstetrics (FIGO) report  (5, 6). Increased morbidity and complications have been specifically demonstrated when surgery and radiotherapy are combined (7, 8). Therefore, a preoperative and noninvasive assessment to predict adverse pathologic factors is of great importance to optimize a treatment plan to lower the incidence of post-treatment morbidity and improve the quality of life.

Lymph-vascular space invasion (LVSI) is defined as the presence of carcinoma cells within the lymphatic and/or blood vessels (9). LVSI has been widely recognized as a risk factor in cervical cancer. Previous studies suggest that the presence of LVSI predicts risk of nodal metastasis which has direct impact on the prognosis of cervical cancer patients (10). NCCN guidelines recommend that adjuvant pelvic radiation ± chemotherapy should be taken by patients, when “Sedlis Criteria” was met (**Table 1**). The tumor size and the depth of invasion could be predicted preoperatively with a good performance, while studies on LVSI are much less and the predictive performance of LVSI is unsatisfying (11, 12). As the important but missing link for patients to select an appropriate treatment, the accurate prediction of LVSI before surgery is urgent in clinical practice.

**Table 1:** Sedlis Criteria for external pelvic radiation after radical hysterectomy in node-negative, margin-negative, parametria-negative cases.

LVSI	Stromal Invasion	Tumor Size (cm) (determined by clinical palpation)
+	Deep 1/3	Any
+	Middle 1/3	≥2
+	Superficial 1/3	≥5
–	Middle or deep 1/3	≥4

LVSI, Lymphovascular space invasion.

Radiomics analysis can extract a large number of quantitative features from medical images and convert them into various high-dimensional data, providing information on tumor heterogeneity (13–15). Successful evaluation and predictive capabilities have been achieved in a variety of challenging clinical analysis by developing appropriate model refinement features. Radiomic analysis can be used to diagnose diseases such as lymph node metastatic status of rectal cancer, bladder cancer, and breast cancer cost-effectively and non-invasively (16–20). T2 weighted MRI and diffusion-weighted imaging are also widely used in the staging of localized cervical cancer.

Therefore, the objective of the study was to build and evaluate a radiomics-based nomogram for improving the predictive performance of the LVSI in cervical cancer.

## Materials and Methods

### Patient

**Figure 1:**
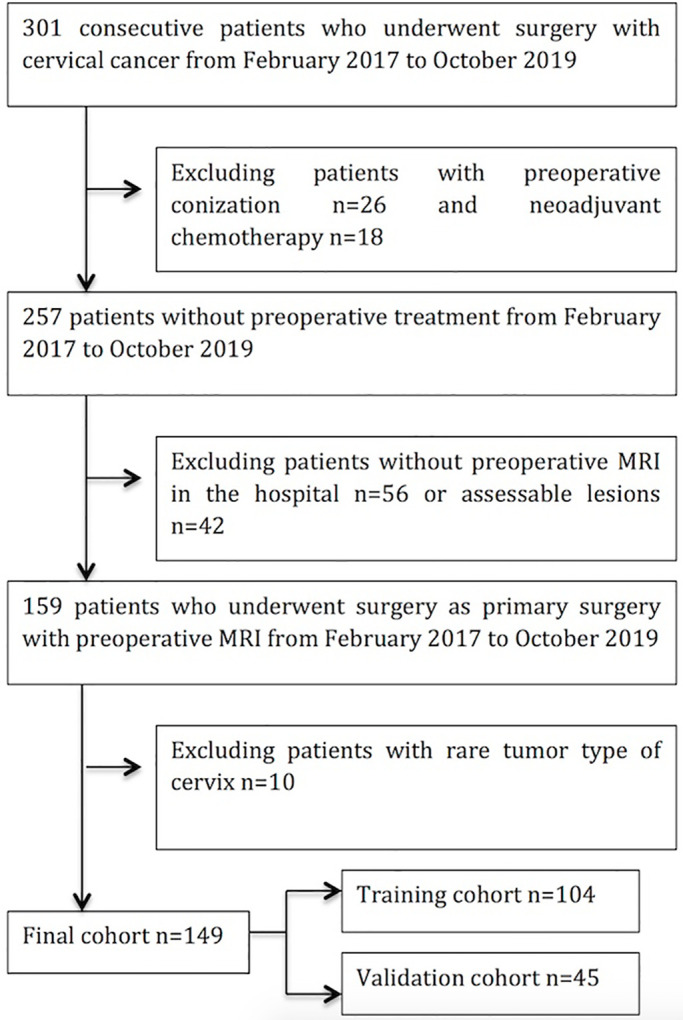
Flow diagram of the study enrollment patients.

Our hospital ethics committee approved this retrospective study and patient informed consent was obtained. 301 consecutive patients with cervical cancer with biopsy-proven cervical carcinoma received initial treatment in our hospital between February 2017 and October 2019 were enrolled in our study. The inclusion criteria were as follows: (i) patients who underwent surgery with pathologically confirmed cervical cancer; (ii) All patients who received a pretreatment MRI scan; (iii) primary cervical lesions were visible on sagittal T2WI. The exclusion criteria were as follows: (i) history of preoperative therapy (neoadjuvant chemotherapy, radiotherapy, or conization); (ii) absence of preoperative MR in this hospital (iii) insufficient image quality; (iv) rare types of cervical tumor. MRI scans were reviewed by two radiologists with 7 and 5 years of experience. The patient selection process is shown in **Figure 1**.

### MRI Acquisition Protocol

All patients recruited in this study were examined using the 3.0T MRI scanner (Achieva 3.0 T, Philips, Amsterdam, Netherlands) equipped with a 16-channel abdominal coil. The MR scan covered the area from the superior edge of the iliac crest to the lower edge of the pubic symphysis. The parameters of some 3.0T MRI sequences were as follows: axial fat-suppressed turbo spin-echo (TSE) T2-weighted images (TR/TE: 4854/85ms, FOV = 300 × 300 mm; matrix = 232 × 171; slice thickness/gap: 5/1mm; NEX = 2).

### Image Segmentation

**Figure 2:**
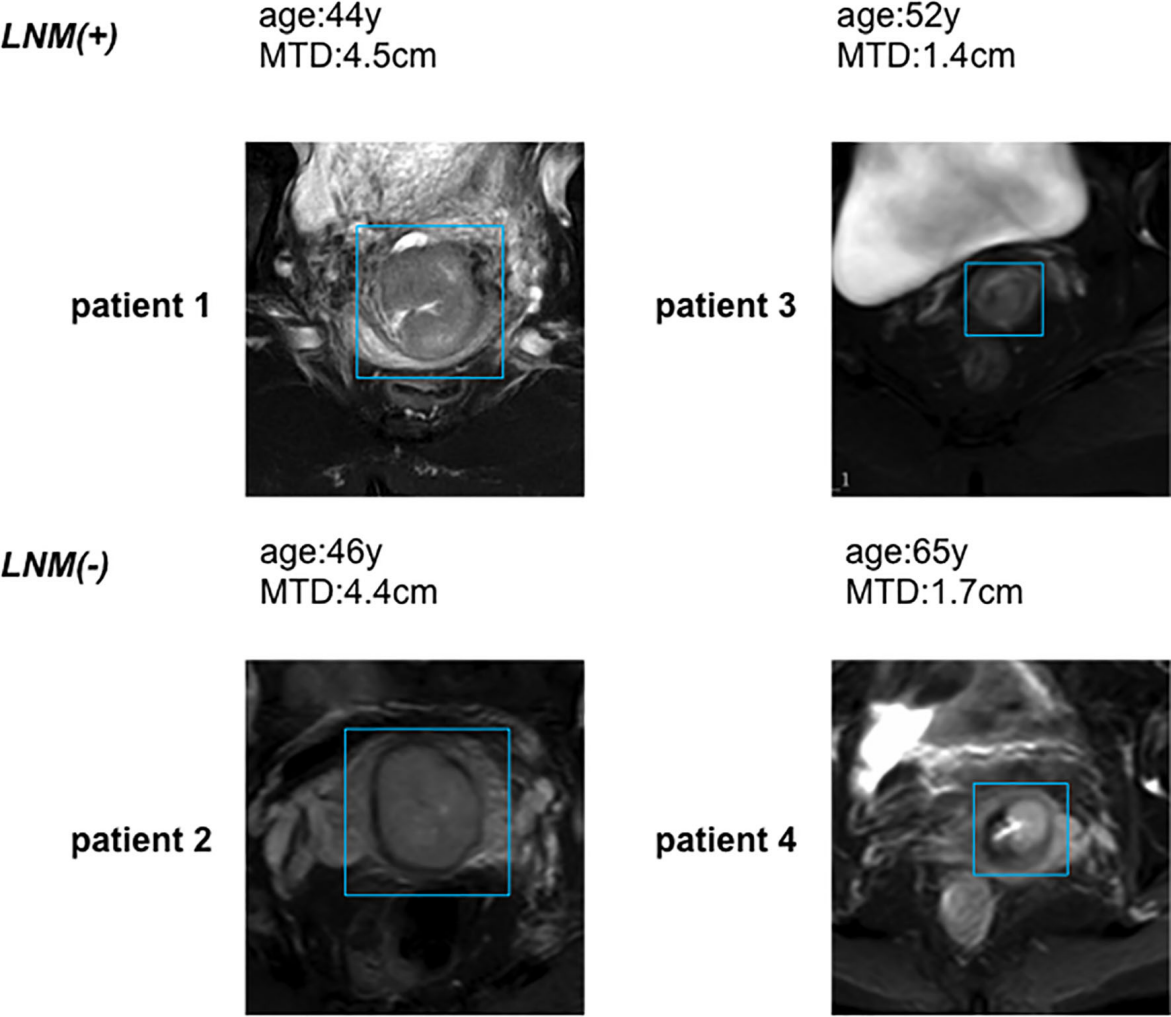
Representative MRI images in LVSI(-) and LVSI(+) patients.

**Figure 3:**
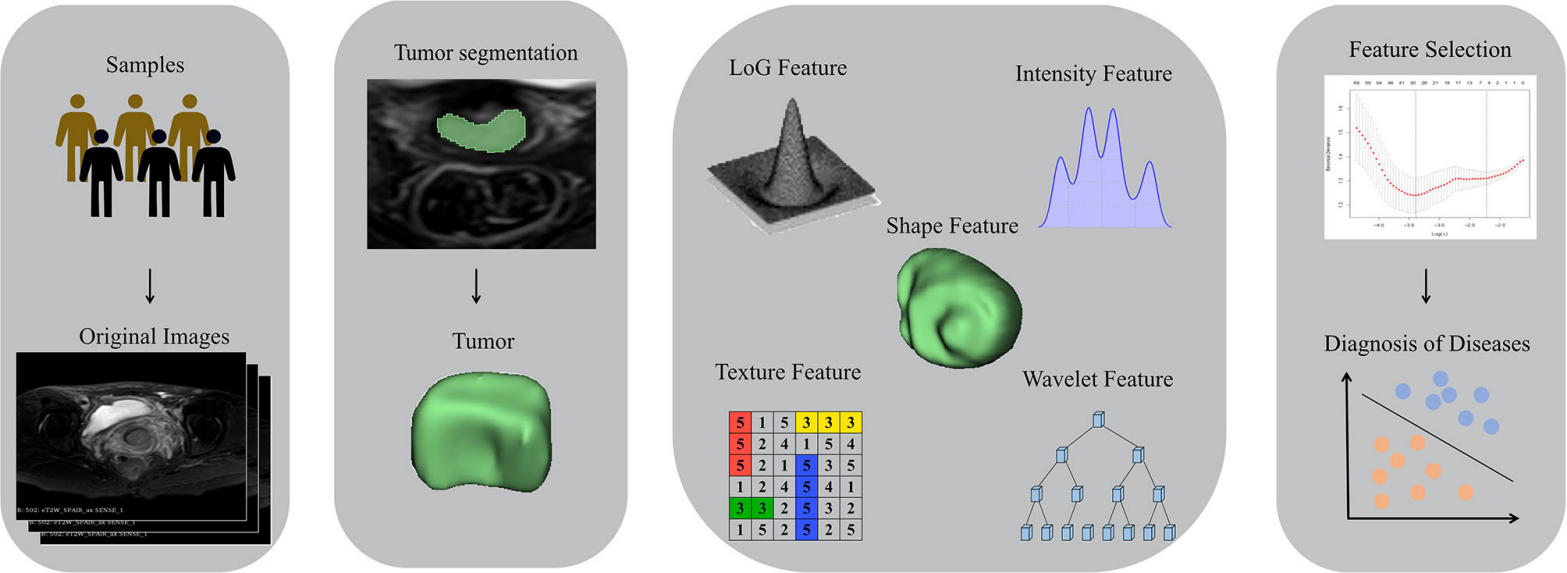
Workflow of radiomics analysis. T2WI images were collected. Regions of interests (ROI) of the tumor lesions were manually delineated. Radiomics features were extracted. Discriminative features were selected by the LASSO regression model. Prediction model was constructed by radiomics signature; ROC curves were performed for further statistical analyses.

3D slicer software was explored for three-dimensional manual segmentation (open-source software; https://download.slicer.org/). All manual segmentations of the tumor tissues on axial T2WI were done by a radiologist who had 5 years of experience in gynecological MR imaging. And each segmentation was validated by a senior radiologist, who had 7 years of experience. The representative images of lesions are in **Figure 2**. The workflow of the radiomics analysis is presented in **Figure 3**.

### Radiomic Feature Extraction

After manual segmentation of the tumor was completed, open-source software called pyradiomics was used to complete the extraction of the radiographic features of the tumor. Standardized operations on T2WI images were used to obtain a standardized normal-distribution image distribution. On T2WI of the tumor, we extracted 1682 radiological features from seven image types. These features are used to quantify tumor size (e.g., volume), shape (e.g., circumference, diameter), grayscale co-occurrence matrices (e.g., energy, contrast, entropy), grayscale run-length matrices, and grayscale dependency matrices. All features are implemented using python 3.7.

### Radiomics Feature Selection and Development of Radiomics Models

Although radiographic features reflect the characteristics of the tumor tissue from different perspectives, not all information is relevant to cervical cancer. We used the LASSO algorithm as a means of our feature extraction to select non-zero coefficients in the training data by 10-fold crossover regression to obtain the desired radiomics features. Chalkidou et al. proposed that a minimum of 10 to 15 observations per predictor variable is required to build a reasonably stable model. The variables need to be modeled in a reasonably stable manner. Therefore, recursive feature elimination (RFE) features are selected to avoid further overfitting. Radiomics Model Building.

After feature selection, we use a support vector machine (SVM) model to predict sample types. This SVM model uses a linear kernel and the regularization parameter C to determine the best possible effect of the model. The C value that maximizes the AUC is used as the best parameter for the training group.

### Development of Clinical Model

The stromal invasion depth, MTD and FIGO stages showed a significant difference (p < 0.05) between LVSI positive and LVSI negative groups in either the training cohort or the validation cohort in **Table 2**. The stromal invasion depth, MTD and FIGO stages were studied to build the clinical predictive model. SVM model was used to predict sample types.

**Table 2:** Characteristics of involved patients.

Characteristics	Training cohort (n = 104 )			Validation cohort (n = 45 )			P*
	LVSI (+)	LVSI (-)		LVSI (+)	LVSI (-)		
	(n = 45 )	(n =59 )	P	(n = 22 )	(n = 23 )	P	
Age, years			0.114			0.205	0.322
Mean	45	48		44	46		
Range	37-53	40-56		35-53	42-50		
FIGO Stage(N,%)			0.878			0.027	0.698
IA	1	0		0	0		
IB	28	38		9	17		
IIA	16	21		13	6		
MTD			0.042			0.314	0.366
≤4 cm	27	47		15	19		
>4 cm	18	12		7	4		
Histology(N,%)			0.071			0.203	0.283
SCC	41	44		19	21		
AC	3	13		1	2		
ASC	1	2		2	0		
Stromal Invasion			<0.0001			<0.0001	0.597
Deep 1/3	28	17		13	6		
Middle 1/3	14	16		6	4		
Superficial 1/3	3	26		3	13		
p-LN status			<0.0001			<0.0001	0.509
Positive	23	3		9	0		
Negative	22	56		13	23		

P is derived from the chi-squared test or Fisher’s exact test between patients with and without LVSI in the training and validation cohort respectively. P* represents the difference of each clinicopathological variable between the training and validation cohort. MTD, maximal tumor diameter; LVSI, lymphovascular invasion; SCC, squamous cell carcinoma; AC, adenocarcinoma; ASC, adenosquamous carcinoma; p-LN status, pathological lymph node status.

### Development of the Radiomics-Based Nomogram

The combined model was built using the logistic regression method with forward stepwise selection. The radiomics signature and clinical risk factors were used in the combined model. To make the model a more easy-to-use tool for preoperative prediction of the LVSI status, the combined model was visualized as the radiomics-based nomogram. The formula of the radiomics signature of the final radiomics model is shown in **Supplementary S1**.

### Assessment of Predictive Models

The performances of these predictive models were first assessed on the training cohort and then validated on the validation cohort using receiver operating characteristic (ROC) curve. Calibration curve were used to assess the agreement between nomogram prediction probabilities of the LVSI status and actual outcomes. To assess the difference between the radiomics-based nomogram and the clinical model, the DeLong test was performed.

### Statistical Analysis

Statistical analysis was conducted with SPSS 21, R 3.4.1 and python 3.7. The independent-sample t-test was used to compare the mean value on age between different groups. The chi-squared test or Fisher’s exact test was used to evaluate the significance of the categorical variables in the training and validation cohorts, respectively. Two-tailed p-values <0.05 were considered statistically significant. The logistic regression with the LASSO penalty and the SVM model was implemented using python 3.7 in the scikit-learn package.

## Result

### Demographic, Clinical, and Histopathological Characteristics

Of the 301 patients underwent surgery with cervical cancer from February 2017 to October 2019, 26 patients with preoperative neoadjuvant chemotherapy and 18 patients with preoperative conization were excluded; 56 patients and 42 patients were excluded for having no preoperative MRI in the hospital or no assessable lesions on MRI; 10 patients were excluded for rare tumor type of cervix; finally, 149 patients fulfilled the eligibility criteria and were enrolled in following analysis (**Figure 1**).

Patient characteristics are summarized in **Table 2**. There were no significant differences in the clinical characteristics between the training and validation cohorts. The stromal invasion and p-LN status showed a statistical difference between patients with and without LVSI both in the training and validation cohorts, as were shown in **Table 2**.

### Feature Selection, Performance of Clinical Model, and Radiomics Model

**Figure 4:**
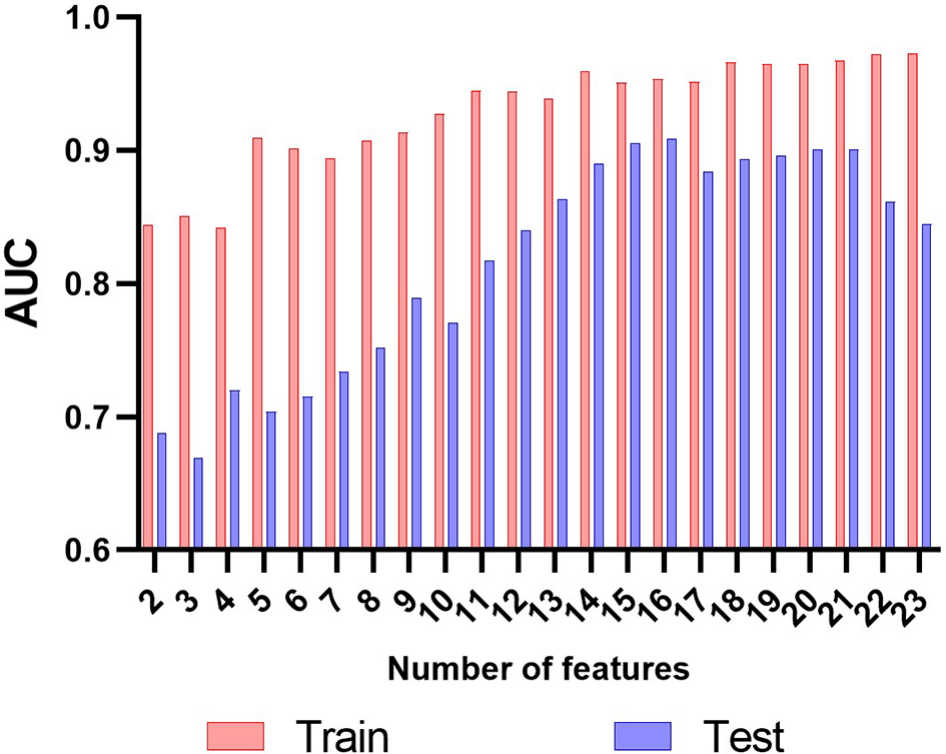
The results of the AUC in radiomics feature selection. The results of the AUC showed that the features at 14 got a good performance both in the training cohort and in the testing cohort.

Thirty features were obtained in the feature extraction of radiomics, and in order to avoid overfitting of the model. We used RFE algorithm to achieve further feature extraction, and to make the selection of feature number more reasonable, AUC, accuracies, sensitivity and specificity were selected as references. The results of the AUC in **Figure 4** showed that the features are more suitable at 14, and the combination performs more compared to other combinations of features between 10 and 15, and other groups of feature combinations are shown in the **Supplementary S2**. Among these extracted features mainly include 2 exp features and 8 wave features, and the details are in **Table 3**.

**Figure 5:**
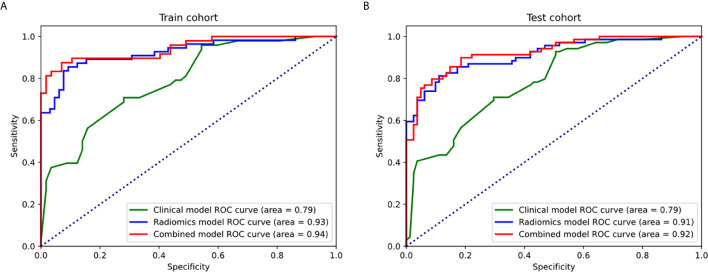
The ROC curves of the clinical model, radiomics models, and combined model in the training cohort **(A)** and testing cohort **(B)**.

Clinical model that combine stromal invasion depth, FIGO stage, and MTD exhibit unsatisfying performance in the training cohort (AUC=0.786, accuracy=70.5%, sensitivity=41.7%, and specificity=94.7%) and the testing cohort (AUC=0.706, accuracy=71.3%, sensitivity=43.4%, and specificity=95.0%). The radiomics model performed much better than the clinical model in both training (AUC=0.925, accuracy=87.5%, sensitivity=83.6%, and specificity=90.7%) and the testing cohort (AUC=0.911, accuracy=84.0%, sensitivity=81.1%, and specificity=86.4%)(**Table 4**). The ROC curves were shown in **Figure 5**.

**Table 3:** Radiomics Screening Features.

No.	Feature
1	original_shape_Sphericity
2	exponential_firstorder_Minimum
3	exponential_glszm_GrayLevelVariance
4	lbp-3D-k_glrlm_RunVariance
5	logarithm_firstorder_Kurtosis
6	square_glcm_MCC
7	wavelet-LHH_gldm_LargeDependenceHighGrayLevelEmphasis
8	wavelet-HLL_firstorder_Energy
9	wavelet-HLL_gldm_DependenceVariance
10	wavelet-HLL_glszm_LargeAreaLowGrayLevelEmphasis
11	wavelet-HLL_ngtdm_Busyness
12	wavelet-HLH_glszm_SmallAreaEmphasis
13	wavelet-HHL_glszm_SmallAreaLowGrayLevelEmphasis
14	wavelet-LLL_glcm_MCC

**Table 4:** Performance of models.

Models	Training cohort				Testing cohort			
	AUC	ACC(%)	SEN(%)	SPE(%)	AUC	ACC(%)	SEN(%)	SPE(%)
Clinical	0.786	0.705	0.417	0.947	0.706	0.713	0.434	0.950
Radiomics	0.925	0.875	0.836	0.907	0.911	0.840	0.811	0.864
Combined	0.943	0.895	0.854	0.929	0.923	0.846	0.84	0.851

### Performance of the Radiomics Nomogram

**Figure 6:**
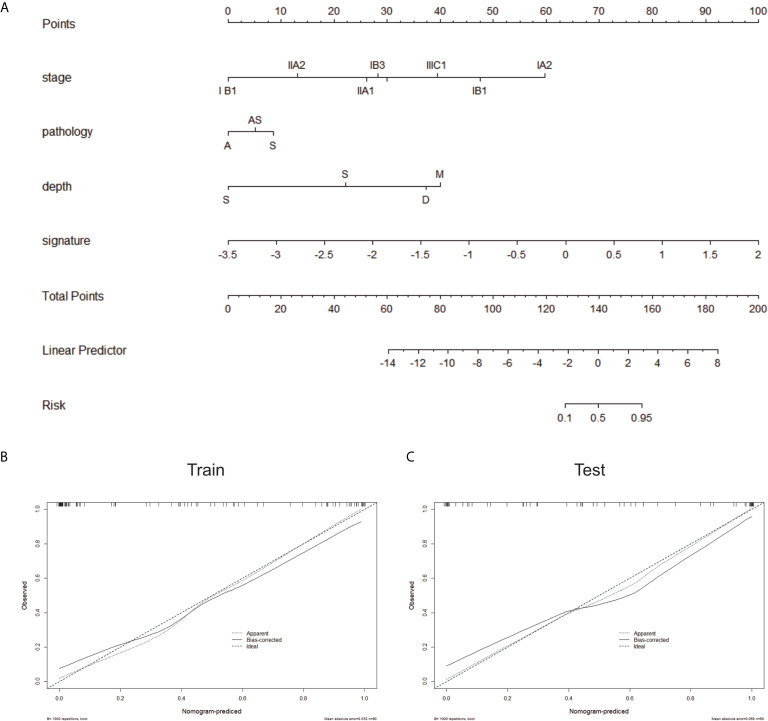
**(A)** The radiomics-based nomogram. Calibration curves in the training cohort **(B)** and validation cohort **(C)**. Closer fit to the diagonal line indicates a better evaluation.

During the development of combined model, tumor stage, tumor infiltration depth and radiomics signature were selected. Compared with the radiomics model and clinical model, the combined model reached the highest AUC in the training cohort (AUC=0.943, accuracy=89.5%, sensitivity=85.4%, and specificity=92.9%) and the testing cohort (AUC=0.923, accuracy=84.6%, sensitivity=84.0%, and specificity=85.1%)(**Table 4** and **Figure 5**). The DeLong tests revealed a significant difference between the clinical model and combined model in the training and validation cohorts (p = 0.012 and 0.038). The radiomics-based nomogram for visualization of the combined model is shown in **Figure 6**. The calibration curves of the radiomics-based nomogram demonstrated satisfactory agreement between the predictive and observational possibility of the LVSI status in both the training and validation cohorts.

## Discussion

Adverse pathologic factors are categorized into high-risk, intermediate-risk, or low-risk. High-risk factors include positive surgical margins, lymph node metastases, and parametrial spread. Patients with any high-risk factor need PORT with chemotherapy (GOG 109 trial) (21). Intermediate-risk factors include tumor size more than 4 cm, lymphovascular invasion, and deep stromal invasion. Patients with any two of three intermediate-risk factors require PORT (22, 23). Many attempts have been made to evaluate adverse pathologic factors at primary staging. MRI and ultrasound can accurately evaluate deep stromal invasion, parametrial spread, and tumor size (24–28). For the detection of pelvic lymph node metastases, PET/CT, PET/MRI, and MRI with USPIO have a better diagnostic performance than conventional MRI, CT, and US, providing noninvasive imaging methods particularly in patients at high risk of metastatic disease (29–31).

As one of the intermediate-risk factors, the presence of LVSI guides treatment selection. For patients with negative margins after cone biopsy and no findings of LVSI, observation may be an option if fertility preservation is desired. For patients with stage IA1 disease with LVSI, conization (with negative margins) plus laparoscopic pelvic SLN mapping/lymphadenectomy is a reasonable strategy (32–35). For early-stage patients receiving radical hysterectomy, both the NCCN guidelines and the 2018 FIGO report recommend that patients need PORT with chemotherapy in the presence of positive lymph nodes and/or surgical margins and/or parametrial involvement (5, 6). NCCN guidelines also recommend that pelvic radiation ± chemotherapy should be taken in nodes-negative cases, when “Sedlis Criteria” was met. The “Sedlis Criteria,” which are intermediate-risk factors used to guide adjuvant treatment decisions, include 1) greater than one-third stromal invasion; 2) capillary lymphatic space involvement; or 3) cervical tumor diameters more than 4 cm (**Table 2**). The reported rate of adjuvant RT following radical hysterectomy and pelvic lymphadenectomy is 10% to 64% (2, 8, 36–40). Thus, a significant proportion of patients undergo treatment by both modalities with potentially compounding morbidities.

Although LVSI status is important in treatment plan guiding, the status of LVSI lacks effective prediction before an operation. While other risk factors have relatively predictive accuracy, which makes LVSI the bottleneck in optimizing treatment. Transvaginal or transrectal ultrasound (TRUS/TVUS) and conventional MRI have a high diagnostic accuracy in tumor size and deep stromal invasion, with reported accuracy 95% and 93%, 93% and 88%, respectively (27–29, 41–43). TRUS/TVUS and MRI with DWI have shown good diagnostic performance in a parametrial invasion, with a reported accuracy of 87%-99% and 78%-99%, respectively (44–46). PET/MRI and radiomics based on conventional MRI yielded high diagnostic accuracy in lymph node metastases (47–49). Preoperative imaging had a relatively high diagnostic efficacy in other risk factors described above, however, few studies concentrated on the preoperative prediction of LVSI with diagnostic imaging. In the retrospective research by Chen et al., it was shown that Gross tumor volume and the maximum diameter of resettable cervical cancer at MRI demonstrated capability in predicting LVSI, with an AUC of 0.700 (50). Yang et al. reported that the Mini-ADC value appears to be a simple and effective tool for the prediction of LVI status in invasive CC with an AUC of 0.885 (51). To enhance the diagnostic performance, radiomics was utilized in the prediction of LVSI. Li et al. found that the radiomics showed discrimination between LVSI and non-LVSI groups. The AUC was 0.710 in the training cohort and 0.633 in the validation cohort (52). In Wu et al.’s research, radiomics analysis of multiparametric MRI evaluates the presence of LVSI. The area under AUC of anatomical, diffusion, and permeability parameters in discriminating the presence of LVSI ranged from 0.659 to 0.814 (53). Unsatisfactory diagnostic accuracy was reported according to these previous studies. According to “Sedlis criteria”, patients were recommended to PORT when two of three immediate-risk factors exist. When the tumor size and depth of stromal invasion could be evaluated with high accuracy, the accurately assessment of LVSI seems more urgent.

Medical imaging acts as a non-invasive phenotypic feature, and the radiographic features of it are extracted quantitatively, which may feedback into the heterogeneity between samples. In this study, the acquired 4 LBP and 18 wavelet features were included in the radiomics features of T2. The wavelet transform, as a filtering algorithm, can perform local amplification of spatial information by highlighting certain information features through the transform. Therefore, certain characteristics of the tumor can be amplified, for example, the tumor’s partial state information and the degree of gray level dependence can be further highlighted. Furthermore, the LBP operator, as an algorithm for describing the local texture features of an image, can not only perform the task of special diagnosis extraction but also highlight the texture features of the image. Besides, some of the remaining features are also done to some extent to highlight the image texture features. For example, logarithm_firstorder_Kurtosis by measuring the kurtosis of the median value distribution of the image ROI, a low kurtosis can make the texture sharper. Wang et al. found that a non-Gaussian-based diffusion-weighted model helps to initially differentiate pelvic cancer grade as well as stage by kurtosis. And Tapera et al. found that pelvic cancer spherical again presented significant in Bartlett’s sphericity tests, p<0.001. With the quantitative radiomics analysis, it is possible to use high-dimensional image information as the basis of disease diagnosis. MRI-based radiomics showed high AUCs in differentiating LVSI (+) from LVSI (-) in the training cohort and validation cohort. Our results suggest that the radiomics analysis of a T2WI map could identify the status of LVSI in cervical cancer with sensitivity and specificity of 0.875 and 0.836 in the training cohort, of 0.811 and 0.864 in the testing cohort, respectively. The AUC of the combined model was 0.943 in the training cohort and 0.922 in the testing cohort, which is the best in all published researches of LVSI in cervical cancer. Combined with the precise prediction of LVSI, tailored treatment could be received by early-stage cervical cancer patients.

There are some limitations to our study. This study was a single-center retrospective study. The retrospective nature of this study may be a reason for potential bias. Secondly, although the number of enrolled patients in this study is the highest in published articles on radiomics-analysis of LVSI on cervical cancer, the available patient number was still low. Results from our database should be supplemented with further prospective and external validation by a larger sample size.

In conclusion, the radiomics-based combined model showed a good performance for predicting LVSI of cervical cancer. The radiomics-based nomogram represents an effective tool for the preoperative individualized prediction of LVSI. This would aid the selection of the optimal therapeutic strategy and clinical decision-making for individuals.

## Data Availability Statement

The original contributions presented in the study are included in the article/**Supplementary Material**. Further inquiries can be directed to the corresponding author.

## Ethics Statement

The studies involving human participants were reviewed and approved by Ethics Committee of West China Hospital, Sichuan University. The patients/participants provided their written informed consent to participate in this study.

## Author Contributions

WD and ZL designed the methods. XuX and YW collected the data and segmented images. WD, YW and ZL analysed the data. WD and DL wrote and finalized the paper.

## Conflict of Interest

The authors declare that the research was conducted in the absence of any commercial or financial relationships that could be construed as a potential conflict of interest.
