# Supplementary material for: Preoperative Prediction of Lymphovascular Space Invasion in Cervical Cancer With Radiomics –Based Nomogram
Source: Front Oncol. 2021 Jul 12;11:637794. doi: 10.3389/fonc.2021.637794 (PMC8311659; doi:10.3389/fonc.2021.637794)
Supplement: Supplementary file 2 [file DataSheet_2.docx]

**Supplementary S1: The formula of the radiomics signature**

$$Radimocs score=-1\times b+\sum_{i=1}^{N} (\boldsymbol{sv}_{\boldsymbol{i}}\boldsymbol{\cdot x})$$

Where

$b$ being the intercept,

$N$ being the number of support vectors,

$\boldsymbol{sv}_{\boldsymbol{i}}$ being the $i$^th^ support vector,

$x$ being the new data, and

$$x=[T1\_Edema\_Coif2\_glrlm\_RP, T1\_Edema\_Coif1\_glszm\_GLN, T1\_Tumor\_Coif2\_median, T2\_Tumor\_Coif8\_glszm\_SAE,ADC\_Tumor\_Coif1\_glszm\_SAE, ADC\_Tumor\_Coif4\_glszm\_GLV]$$

$\boldsymbol{\cdot}$ being the dot product.

This radiomics score was used to predict whether a new case should be mutated or wild-type IDH, based on the cut-off value shown in Table 2. To get the probability of mutated IDH1 $Pi$, we could use the following formula:

$$Pi=\frac{1}{1+e^{-x}}$$

Where $x$ is the radiomics score.
